# Effectiveness of educational intervention in improving physical activity and nutritional performance among pregnant women: a pre-post quasi-experimental study using health belief model

**DOI:** 10.3389/fgwh.2024.1471957

**Published:** 2024-12-23

**Authors:** Ali Khani Jeihooni, Fatemeh Razmjouie, Hanieh Jormand, Fariba Sedghi Jahromi, Pooyan Afzali Harsini, Amirhossein Kamyab, Farzaneh Ghaleh Golab

**Affiliations:** ^1^ Nutrition Research Center, Department of Public Health, School of Health, Shiraz University of Medical Sciences, Shiraz, Iran; ^2^ Department of Health Promotion and Aging, School of Health, Shiraz University of Medical Sciences, Shiraz, Iran; ^3^ Urology and Nephrology Research Center, Hamadan University of Medical Sciences, Hamadan, Iran; ^4^ Kermanshah University of Medical Sciences, Kermanshah, Iran; ^5^ Faculty of Medicine, Fasa University of Medical Sciences, Fasa, Iran; ^6^ Student Research Committee, School of Health Management and Information Sciences, Shiraz University of Medical Sciences, Shiraz, Iran

**Keywords:** physical activity, pregnant women, nutrition, education, health belief model (HBM)

## Abstract

**Background:**

Physical activity and proper nutrition during pregnancy are crucial for maternal and fetal health. However, many pregnant women fail to meet recommended guidelines. This study aimed to assess the effectiveness of an educational intervention based on the Health Belief Model (HBM) in improving these behaviors.

**Methods:**

A pre-post with control quasi-experimental study was conducted among 200 pregnant women (100 experimental and 100 control) in Shiraz, Iran. The intervention group attended eight weekly 50-minute educational sessions based on the HBM. Physical activity and nutritional performance were assessed using validated questionnaires at baseline and three months post-intervention. Data were analyzed using paired and independent *t*-tests, with effect sizes and 95% confidence intervals reported.

**Results:**

The intervention significantly improved physical activity (mean score: 29.25 ± 4.42 vs. 12.28 ± 4.36, *p* < 0.001) and nutritional performance across all food groups (*p* < 0.001) in the experimental group compared to the control group. Key constructs of the HBM, including perceived sensitivity, severity, benefits, self-efficacy, and cues to action, showed significant increases with notable effect sizes and 95% confidence intervals, while perceived barriers significantly decreased (*p* < 0.001).

**Conclusions:**

The educational intervention based on the HBM effectively improved physical activity and nutritional performance among pregnant women. Integrating such programs into routine prenatal care is recommended, with emphasis on personalized counseling, regular follow-ups, and spousal involvement to sustain behavioral changes and promote maternal and fetal health.

## Background

Although pregnancy is a time of physical, hormonal, and humoral changes preparing the mother's body for childbirth and breastfeeding, providing vital nutrients to the fetus, this biological phenomenon is regarded as a window into the mother's and child's future health (1). Pregnant women take better care of themselves, are more receptive to new ideas, and are likelier to adopt healthy lifestyle changes; therefore, recommendations to increase physical activity (PA) and consume a balanced diet during pregnancy and after delivery are beneficial to both mother and baby (2).

Numerous health advantages have been linked to regular PA, including increased physical fitness, better mental health, a lower risk of developing chronic diseases, and a lower risk of passing away (3). Pregnant women are now recommended to exercise vigorously for 75 min or moderately for 150 min each week, according to the World Health Organization (WHO) (4); less than 15% of pregnant women meet the minimum recommendation (5). Over the past three decades, the frequency of pregnancy problems such as gestational diabetes, preeclampsia, gestational hypertension, and neonatal macrosomia has increased considerably, most likely due to the rise in maternal fat and insufficient PA (6, 7).

It is important to note that pregnant women and their fetuses require proper nutrition and that their dietary habits and nutritional status greatly influence pregnant women's health (8). Sufficient and correct nutrition during pregnancy and supporting adequate fetal growth prepare the mother for breastfeeding, particularly in the first six months (9). Health professionals and politicians agree that pregnant women should consume a balanced diet to ensure a healthy pregnancy. Because the mother is the primary source of nutrients for the fetus during pregnancy, her diet and nutritional storage are likely to impact the newborn's nerve development during the intrauterine period. Micronutrients, such as vitamins and minerals, help produce and distribute neurotransmitters, whereas macronutrients (carbohydrates, proteins, and fats) are the building blocks for total brain growth (10).

A valid indicator of nutritional status control is maternal weight gain during pregnancy (11). If the recommended amount is less than normal, it results in maternal anemia, low birth weight, and neurological abnormalities in the infant. If more than advised, it may lead to gestational hypertension, diabetes, increased BMI, and a large baby's birth (12, 13). Constipation, indigestion, and urinary tract infections are a few pregnancy-related issues that can be prevented and treated with a healthy diet and regular exercise (14). Health authorities have recommended that all pregnant mothers follow a healthy, forward-looking diet (15). Because expecting mothers are concerned about their health and the health of their unborn children, they pursue health information. Consequently, assessing their knowledge and attitudes during this period is essential in planning for dietary adjustments (16).

One of the most important strategies in development programs to improve nutrition and PA is to increase expecting mothers’ knowledge through implementing educational interventions to prevent nutritionally-related disorders (12). Consequently, the present study aimed to determine the effect of educational intervention based on the Health Belief Model (HBM) on promoting PA and NP of pregnant women in the suburbs of Shiraz, Iran. The HBM facilitates behavioral modification (17). Key constructs of this model include perceived sensitivity (one's sensitivity to a particular behavior), perceived severity (one's beliefs about the severity of a particular behavior), perceived benefits (one's perception of the advantages of adopting a particular behavior), perceived barriers (one's perception of the challenges standing in the way of any health behavior), cues to action (stimuli that prompt a decision and urge to act), and self-efficacy (one's confidence in their capacity to carry out a behavior successfully) (18).

Many studies have been conducted on pregnant women's PA and nutrition based on HBM. In this regard, studies by Riazi et al. (19) and Diddana et al. (20) showed that nutrition education based on HBM significantly increased nutrition knowledge and dietary intake practices among pregnant women. In a study by Pathirathna et al. (21), light to moderate PA during pregnancy was found to be preventive of low birth weight in addition to premature births, intrauterine growth restriction, and low birth weight. Another study showed that HBM-based education increased health-related beliefs in pregnant women, which in turn led to an increase in their level of physical activity during pregnancy (22). The HBM, an individualistic framework intended to change people's knowledge about certain health practices, is the framework used in the present study. The present study aimed to determine the effect of educational intervention based on the Health Belief Model (HBM) on promoting PA and NP of pregnant women in the suburbs of Shiraz, Iran.

## Methods

This pre-post with control quasi-experimental study included 200 pregnant women (100 in the experimental group and 100 in the control group) referred to health centers in the suburbs of Shiraz. The sample size was calculated based on previous studies, ensuring 80% power and a 95% confidence interval (12, 23). Four health centers were randomly selected: two for the experimental group and two for the control group. Eligible participants, in the 8th to 14th week of pregnancy, were identified from health center records and alternately assigned to the experimental and control groups. Study objectives were explained to participants, and informed consent was obtained.

The sample size for this study was calculated based on previous studies that assessed similar educational interventions using the HBM. The calculation aimed to achieve 80% statistical power and a 95% confidence level to detect significant differences between the intervention and control groups in terms of physical activity and nutritional performance. A minimum detectable effect size of 0.5 was assumed, considering expected changes in key outcome variables. Using these parameters, the required sample size was determined to be 200 participants (100 in each group) to account for potential attrition and ensure adequate statistical reliability.

### Inclusion/exclusion criteria

Participants were eligible if they were literate, in the 8th to 14th week of pregnancy, and had no history of chronic conditions such as diabetes, hypertension (including eclampsia or preeclampsia), heart disease, or gastrointestinal issues. Exclusion criteria included the occurrence of complications during the current pregnancy, such as bleeding, bladder rupture, eclampsia, preeclampsia, gestational diabetes, miscarriage, or adherence to a strict diet.

### Data collection

Data collection tools included demographic, HBM, PA, and NP questionnaires validated in previous studies (18, 23–26).

#### Demographic questionnaire

This collected information on age, marital status, education, occupation, BMI, income, place of residence, and spouses’ education and occupation to identify baseline characteristics of participants.

#### The health belief model questionnaire

The HBM questionnaire assessed key constructs including knowledge (15 questions), perceived sensitivity (8 questions), perceived severity (7 questions), perceived benefits (8 questions), perceived barriers (7 questions), self-efficacy (8 questions), and cues to action (6 questions). Responses were scored on a 5-point Likert scale ranging from “strongly disagree” to “strongly agree,” reflecting the participants’ attitudes, beliefs, and confidence in adopting health-related behaviors. The reliability and validity of the this questionnaire were established in previous studies. The questionnaire demonstrated strong internal consistency, with Cronbach's alpha values ranging from 0.78 to 0.88 for different constructs, including knowledge, perceived sensitivity, perceived severity, perceived benefits, perceived barriers, cues to action, and self-efficacy. Its validity was confirmed through expert review, ensuring content relevance and clarity, as well as through exploratory factor analysis, which supported the questionnaire's construct validity.

#### The physical activity questionnaire

Adapted from the Persian version of Godwin's questionnaire (27, 28), this tool classified physical activities into mild (e.g., walking, yoga), moderate (e.g., brisk walking, tennis, cycling), and intense activities (e.g., judo, long-distance cycling). Activities performed for at least 15 min weekly were scored as +3 (mild), +5 (moderate), or +9 (intense). For example, a participant walking once weekly and performing judo twice weekly would score 9 using the formula: 3 (1 mild activity) +9 (2 intense activities). This allowed for a comprehensive assessment of PA levels over time. The reliability and validity were well established in prior research (27, 28). The questionnaire demonstrated excellent internal consistency, with Cronbach's alpha values ranging from 0.82 to 0.91 across different activity categories (mild, moderate, and intense). Test-retest reliability showed strong correlations (*r* = 0.85), indicating consistency over time. Validity was confirmed through expert review and criterion-related validation, comparing self-reported physical activity levels with objective measures such as accelerometer data, which showed significant correlations (*r* = 0.78).

#### The nutritional performance questionnaire

Nutritional performance was measured using a validated Food Frequency Questionnaire (FFQ) (29, 30). This tool evaluated the frequency of daily, weekly, or no intake of five key food groups: bread and cereals, meat and proteins, fruits, vegetables, and dairy products. Participants’ consumption patterns were analyzed based on the food pyramid's recommended servings for pregnant women. The reliability and validity were confirmed in previous studies (29, 30). The tool exhibited strong internal consistency, with Cronbach's alpha values ranging from 0.80 to 0.87. Test-retest reliability showed high stability over time, with correlation coefficients exceeding 0.85. Its validity was supported by expert panel evaluations for content relevance and by criterion-related validation, demonstrating significant correlations (*r* = 0.81) with dietary intake data obtained from 24-hour dietary recalls.

### Intervention program

The intervention group participated in an eight-week program comprising weekly 50-minute sessions designed to improve PA and NP through both theoretical and practical education. The sessions were conducted by a multidisciplinary team, including a health educator, nutritionist, gynecologist, and sports physiologist, ensuring that the content was evidence-based, comprehensive, and relevant to the needs of pregnant women.

The first four sessions focused on dietary behaviors, emphasizing the importance of the food pyramid and providing participants with a daily monitoring checklist to track their food intake. These sessions also included discussions on the benefits of a balanced diet for maternal and fetal health, identification of barriers to healthy eating through brainstorming, and development of alternative behaviors via group discussions. Participants were actively encouraged to share their experiences and challenges, creating a supportive environment for peer learning and motivation. Personalized feedback was provided to help participants set realistic dietary goals and monitor their progress.

The following four sessions concentrated on PA during pregnancy, covering its physiological benefits, safety considerations, and strategies to incorporate exercise into daily life. Facilitators introduced a variety of activities, including walking, yoga, brisk walking, badminton, cycling, tennis, swimming, and volleyball, all of which were deemed safe for pregnant women. Practical demonstrations were conducted using role-playing, videos, group neuromuscular exercises, and aerobics to ensure participants understood how to perform these activities correctly and safely.

Participants were given the flexibility to choose PA they preferred from the range introduced, allowing for personalization based on their interests, fitness levels, and comfort. However, facilitators provided structured guidance to help participants develop a balanced routine, ensuring that all selected activities met safety standards and aligned with pregnancy guidelines. For each session, facilitators prepared a suggested schedule of activities, but individual customization was encouraged to foster engagement and adherence to the program.

#### Participant documentation

Participants were provided with forms to track their weekly PA and NP. This documentation aimed to promote accountability and reinforce the application of learned behaviors.

#### Spousal involvement

One session included spouses and health center officials, highlighting their roles in supporting healthy behaviors during pregnancy. Educational materials such as pamphlets and CDs reinforced these topics.

#### Support mechanisms

Participants formed groups of 20 for mutual encouragement and were added to WhatsApp groups for ongoing information exchange. Weekly motivational messages were sent, covering topics such as the benefits of PA, precautions during pregnancy, and practical tips (e.g., “PA strengthens abdominal and pelvic muscles and prepares the body for childbirth” or “Avoid sports that cause overheating or excessive sweating”).

### Follow-Up and control group support

Follow-up sessions were conducted one and two months after the intervention to assess progress and provide additional guidance. Control group participants received routine prenatal care during the study. At the end of the study, the control group was provided with the same educational materials as the intervention group. These materials included booklets and pamphlets detailing the importance of balanced nutrition and PA for maternal and fetal health.

### Ethical considerations

Ethical approval for this study was obtained from the Human Research Ethics Committee at Shiraz University of Medical Sciences. Written informed consent was obtained from all participants after explaining the study's objectives, procedures, and their rights, including the right to withdraw at any time without repercussions. To ensure confidentiality, participant information was anonymized, securely stored, and accessed only by authorized research team members. Additionally, measures were implemented to protect the privacy of all data throughout the study.

### Data analysis

Data were analyzed using SPSS 22 software. Statistical methods included Chi-square tests for categorical variables, independent *t*-tests for between-group comparisons, and paired *t*-tests for within-group comparisons. Measures of association, such as mean differences and effect sizes, were reported with 95% confidence intervals. Missing data were managed by excluding incomplete cases from the analysis to ensure accuracy and reliability of the results.

## Results

### Participant flow and recruitmen

**Figure 1:**
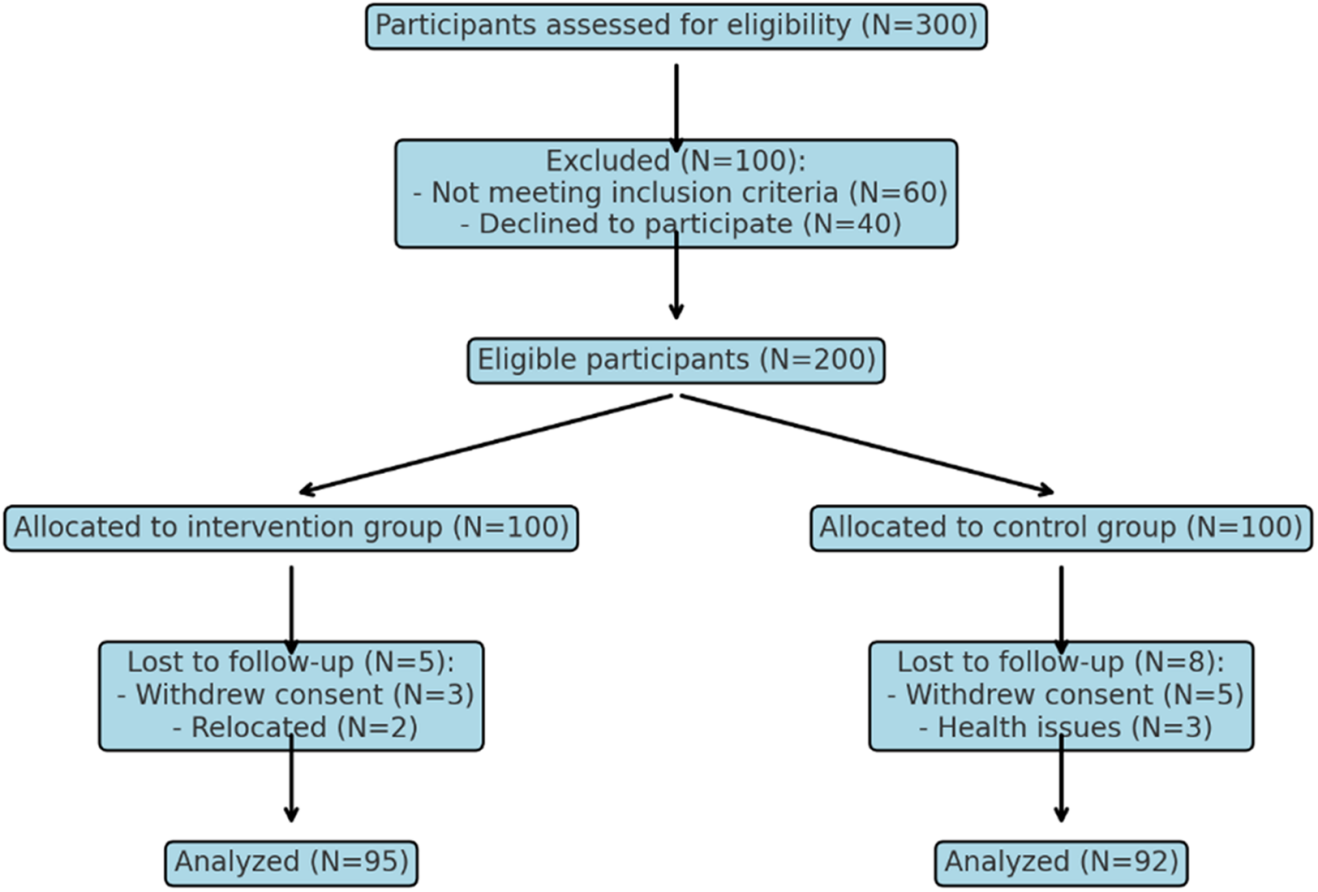
Flowchart of the study.

A total of 200 pregnant women participated, with 100 in the experimental group and 100 in the control group (Figure 1).

### Sociodemographic characteristics of the study participants

The ages of participants ranged from 18 to 45 years. The experimental group had a mean age of 33.6 ± 6.32 years, while the control group had a mean age of 31.94 ± 6.80 years, with no significant difference between the groups (*p* = 0.315). Baseline demographic variables, including marital status, education, occupation, BMI, income, residence, and spouses’ education and occupation, showed no significant differences between the groups (*p* > 0.05; Table 1).

**Table 1:** Comparison of demographic variables of pregnant women participating in the study in two groups of experimental and control.

Variables		Experimental group		Control group		*P* value
		*N*	*P*	*N*	*P*	
Occupation	Household	82	82	86	86	0.359
	Employed	18	18	14	14	
Monthly income	<30 Million Rials	36	36	41	41	0.288
	30–60 Million Rials	47	47	45	45	
	>60 Million Rials	17	17	15	15	
BMI	<19.8	18	18	21	21	0.148
	19.8–26	47	47	50	50	
	26–29	22	22	24	24	
	>30	13	13	5	5	
Education	Primary school	8	8	6	6	0.316
	Secondary school	24	24	22	22	
	High school	51	51	50	50	
	College	17	17	22	22	
Place of residence	Private	48	48	42	42	0.204
	Rent	52	52	58	58	
Spouse's education	Primary school	4	4	9	9	0.295
	Secondary school	20	20	22	22	
	High school	55	55	49	49	
	College	21	21	20	20	
Spouse's occupation	Employed	57	57	54	54	0.337
	Unemployed	43	43	46	46	

### Effectiveness of educational intervention in improving HBM’s constructs

At baseline, both groups had comparable scores for knowledge and HBM constructs. Three months post-intervention, the experimental group demonstrated significant improvements in knowledge and all HBM constructs (perceived sensitivity, severity, benefits, cues to action, and self-efficacy) except perceived barriers (*p* < 0.001). No significant changes were observed in the control group (Table 2).

**Table 2:** Comparison of mean scores of knowledge, perceived sensitivity, perceived severity, perceived benefits, perceived barriers, cues to action, and perceived self-efficacy of pregnant women in the experimental and control groups before and three months after the educational intervention.

Variable	Group	Before the intervention	3 months after the intervention	Paired-*t*-test
Knowledge	Experimental	8.25 ± 1.02	13.10 ± 1.16	0.001
	Control	7.87 ± 1.36	8.06 ± 1.37	0.284
	Independent-*t*-test	0.006	0.001	
Perceived sensitivity	Experimental	18.22 ± 3.33	34.46 ± 3.40	0.001
	Control	20.12 ± 3.14	21.33 ± 3.20	0.208
	Independent-*t*-test	<0.001	0.001	
Perceived severity	Experimental	16.58 ± 3.70	35.21 ± 3.52	0.001
	Control	18.26 ± 3.67	19.68 ± 3.50	0.214
	Independent-*t*-test	0.001	0.001	
Perceived benefits	Experimental	19.18 ± 3.90	33.90 ± 3.75	0.001
	Control	17.93 ± 3.74	18.74 ± 3.81	0.222
	Independent-*t*-test	0.011	0.001	
Perceived barriers	Experimental	23.44 ± 2.93	15.55 ± 2.64	0.001
	Control	24.37 ± 3.68	23.72 ± 3.57	0.196
	Independent-*t*-test	0.223	0.001	
Cues to action	Experimental	15.21 ± 1.25	26.02 ± 1.18	0.001
	Control	14.46 ± 1.29	15.37 ± 1.30	0.229
	Independent-*t*-test	0.180	0.001	
Perceived self-efficacy	Experimental	15.97 ± 3.09	34.03 ± 3.19	0.001
	Control	17.87 ± 3.12	19.26 ± 3.14	0.189
	Independent-*t*-test	0.004	0.001	

### Effectiveness of educational intervention in improving nutritional performance

The intervention also significantly improved the nutritional performance (NP) of the experimental group across all food groups (bread and cereals, meat and protein, fruits, vegetables, and dairy products) compared to the control group, which showed no meaningful changes (*p* < 0.001; Table 3).

**Table 3:** Comparison of the mean score of NP in the experimental and control groups before and three months after the educational intervention.

Variable	Group	Before the intervention	3 months after the intervention	Paired-*t*-test
Bread	Experimental	3.35 ± 1.61	6.77 ± 1.84	0.001
	Control	3.27 ± 1.66	3.59 ± 1.64	0.328
	Independent-*t*-test	0.233	0.001	
Meat	Experimental	1.80 ± 0.68	2.66 ± 0.64	0.001
	Control	1.67 ± 0.57	1.83 ± 0.60	0.043
	Independent-*t*-test	0.254	0.001	
Fruit	Experimental	2.98 ± 0.96	4.08 ± 0.94	0.001
	Control	2.59 ± 0.72	2.74 ± 0.69	0.266
	Independent-*t*-test	0.264	0.001	
Vegetable	Experimental	2.58 ± 1.05	4.11 ± 0.65	0.001
	Control	2.71 ± 0.99	2.83 ± 0.94	0.261
	Independent-*t*-test	0.273	0.001	
Dairy product	Experimental	2.47 ± 0.88	3.97 ± 0.56	0.001
	Control	2.22 ± 0.95	2.68 ± 0.97	0.270
	Independent-*t*-test	0.257	0.001	

### Effectiveness of educational intervention in improving physical activity

Baseline physical activity (PA) scores were similar between groups (*p* = 0.392). Following the intervention, the experimental group exhibited a marked increase in PA levels (mean score: 29.25 ± 4.42 vs. 12.28 ± 4.36, *p* < 0.001), whereas the control group remained unchanged (Table 4).

**Table 4:** Comparison of the mean score of PA of pregnant women in the experimental and control groups before and three months after the educational intervention.

Variable	Group	Before the intervention	3 months after the intervention	*P* value
Physical activity	Experimental	12.28 ± 4.36	29.25 ± 4.42	0.001
	Control	12.80 ± 4.22	12.74 ± 4.26	0.920
	*P* value	0.392	0.001	

## Discussion

Based on the results, this study demonstrated a significant improvement in knowledge, health belief model (HBM) constructs, physical activity (PA), and nutritional performance (NP) in pregnant women from the experimental group three months after the educational intervention. The finding of the present study was in agreement with other studies (31–33) which showed that educational interventions improve the knowledge of pregnant women. Also, the results of a study by Widga and Lewis (34) showed that educational interventions have a positive effect on the awareness of pregnant women, which is consistent with the results of the present study and those of Gamboa et al. (35). However, the results of the present study were not consistent with the results of a study by Ostad Rahmi et al. (36) which may be attributed to methodological variations including different educational methods. According to studies, people's knowledge is more about the preconditions of healthy behavior and its increase seems to be necessary for the occurrence of appropriate health behaviors (37). The increased knowledge is accompanied by an increase in people's attitude change, which causes the replacement of correct behavior (nutritional or physical). Spending time and effort by educators and encouraging pregnant women could decrease barriers and improve knowledge.

Perceived sensitivity increase was consistent with the results of studies by Line (38), Buglar (39), and Sharifi Rad et al. (12). Notably addressing increased symptoms, barriers to seeking medications, and, importantly, increased pregnancy problems for mothers and fetuses could improve the perceived sensitivity in the experimental group. In the present study, the perceived severity in the experimental group increased after the intervention, which is consistent with the result of studies by Tavassoli (40) and Charkazi et al. (41). A pregnant woman's belief about the extent of harm that can be caused as a result of inadequate exercise and proper nutrition could change. At this time, it can be expected that the perceived severity will increase significantly.

The educational intervention's effectiveness in reducing perceived barriers with the findings of previous studies, such as those by Canbulat (41) and Lagampan (42), and enhancing perceived benefits align with findings from studies by Diddana et al. (20) and Alizadeh (42). According to the HBM, an increase in perceived threats (perceived sensitivity + severity) is associated with an increase in perceived benefits and a reduction in perceived barriers to health-related behaviors, which is consistent with the results of studies by Downs and Hausenblas (43).

Cues to action refer to stimuli that prompt a decision and urge to act. In this study, cues to action in the experimental group significantly increased after the intervention, which is consistent with the results of studies by Demilew et al. (44), Mekonnen et al. (45), and Permatasari et al. (46). Cues to action seem to rise from social influence and prior experiences of individuals, both of which help change (47). So, applying change strategies such as a motivating partner or other life influencer helps increase the cues to action in the experimental group.

The perceived self-efficacy in the experimental group showed a significant increase after the educational intervention. Our results were consistent with the results of studies by Abood et al. (48), Abdolaliyan et al. (25), and Ferranti et al. (49). Ferranti et al. indicated that improving self-efficacy is an important part of the interventions. Prescription for home-based exercise through communication intervention leads to increased self-efficacy. Also, verbal persuasion by health staff was applied to increase self-efficacy in the experimental group. Pregnant women make changes if they understand that their current situation can have serious consequences for their health and the fetus. Pregnant women's perceived self-efficacy reflects confidence in their capacity to adopt a new health behavior (50).

In the present study, the mean score of NP across the five food groups significantly increased in the intervention group. The results of a study by Emmaet et al. (51) showed a significant increase in the knowledge of pregnant women about omega-3-rich food sources and their benefits in pregnancy, which led them to increase their fish intake significantly and actively buy omega-3-rich products. Also, the results of studies by Chawla et al. (52) and Jing et al. (53) showed the positive effect of education on better NP.

In general, the results of our study showed the effectiveness of educational intervention in improving knowledge, PA, and NP of the subjects. Recommendations by the nutritionists, gynecologists, and other experts and staff involved in the study have proven to be the most important cues to action. The purpose of education was to provide information and enable the audience to evaluate their thoughts, beliefs, and behaviors concerning information and then decide on the changes they need to make.

On the other hand, in terms of PA, our study showed a significant improvement in PA among the experimental group, which is in line with studies by Broberg et al. (54) and Chan et al. (55) who emphasized the mental and physical health benefits of PA during pregnancy. Also, the results of a study by Sheffield et al. (56) showed that women who were more physically active during pregnancy had significantly reduced anxiety symptoms. Muñóz et al. (57) and de Jersey et al. (58) stated that a targeted health education program benefits mothers and their children significantly. Adherence to an exercise program is also influenced by factors such as pre-pregnancy exercise habits, socio-cultural status, equality, and the insistence of healthcare workers to do PA for pregnant women. Notably, in the present study, providing daily regular exercise, delivering a checklist for building scheduled workouts, daily PA, monitoring research performance, and applying other mentioned strategies could increase PA in pregnant women.

### Limitations

This study had several limitations. A key limitation inherent to the quasi-experimental design was the lack of randomization, which may introduce selection bias and limit the ability to establish causal relationships. Additionally, self-reporting of nutritional behavior and PA could lead to information bias, despite efforts to mitigate this by providing participants with clear reporting guidelines. The COVID-19 pandemic further impacted the study by affecting caregiver availability, participant coordination, and attendance at intervention sessions. While the intervention showed improvements in PA and NP, the levels achieved remained below recommended guidelines. Future studies should incorporate strategies to address these limitations, such as employing randomized controlled designs where feasible, using objective measures for PA and NP, and designing interventions aimed at meeting guideline-recommended levels of PA and NP.

## Conclusion

The present study demonstrated the effectiveness of an educational intervention based on the HBM in improving PA and NP among pregnant women. The intervention significantly enhanced knowledge, perceived sensitivity, severity, benefits, self-efficacy, and cues to action, highlighting its potential for broader application.

To enhance generalizability, similar interventions can be adapted for diverse populations by tailoring content to cultural, socioeconomic, and healthcare contexts. Implementing such programs in urban, rural, and underserved settings could further validate their effectiveness and ensure equitable access. Additionally, integrating these interventions into existing prenatal care frameworks can facilitate wider reach.

Actionable recommendations include allocating more time to personalized counseling during prenatal visits to emphasize the importance of PA and balanced nutrition. Regular follow-up sessions and the use of tracking tools, such as weekly monitoring forms, can sustain motivation and ensure adherence over time. Spousal involvement should be encouraged to create a supportive home environment for behavioral change. Furthermore, health and nutrition programs should leverage mass media and community platforms to raise awareness and promote the significance of PA and proper nutrition during pregnancy. Finally, future initiatives should consider involving multidisciplinary teams to provide comprehensive support for pregnant women.

## Data Availability

Data is available from the corresponding author on request.
